# Influence of residual fat signal on diffusion kurtosis MRI of suspicious mammography findings

**DOI:** 10.1038/s41598-020-70154-3

**Published:** 2020-08-06

**Authors:** Anna Mlynarska-Bujny, Sebastian Bickelhaupt, Frederik Bernd Laun, Franziska König, Wolfgang Lederer, Heidi Daniel, Mark Edward Ladd, Heinz-Peter Schlemmer, Stefan Delorme, Tristan Anselm Kuder

**Affiliations:** 1 Department of Radiology, German Cancer Research Center (DKFZ), Heidelberg, Germany; 2 Department of Medical Physics in Radiology, German Cancer Research Center (DKFZ), Im Neuenheimer Feld 280, 69120 Heidelberg, Germany; 3 Faculty of Medicine, University of Heidelberg, Heidelberg, Germany; 4 Junior Group Medical Imaging and Radiology – Cancer Prevention, German Cancer Research Center (DKFZ), Heidelberg, Germany; 5 Institute of Radiology, University Hospital Erlangen, Friedrich-Alexander-Universität Erlangen-Nürnberg (FAU), Erlangen, Germany; 6 Radiological Clinic at the ATOS Clinic Heidelberg, Heidelberg, Germany; 7 Radiology Center Mannheim (RZM), Mannheim, Germany; 8 Faculty of Physics and Astronomy, University of Heidelberg, Heidelberg, Germany

**Keywords:** Breast cancer, Medical research

## Abstract

Recent studies showed the potential of diffusion kurtosis imaging (DKI) as a tool for improved classification of suspicious breast lesions. However, in diffusion-weighted imaging of the female breast, sufficient fat suppression is one of the main factors determining the success. In this study, the data of 198 patients examined in two study centres was analysed using standard diffusion and kurtosis evaluation methods and three DKI fitting approaches accounting phenomenologically for fat-related signal contamination of the lesions. Receiver operating characteristic curve analysis showed the highest area under the curve (AUC) for the method including fat correction terms (AUC = 0.85, *p* < 0.015) in comparison to the values obtained with the standard diffusion (AUC = 0.77) and kurtosis approach (AUC = 0.79). Comparing the two study centres, the AUC value improved from 0.77 to 0.86 (*p* = 0.036) using a fat correction term for the first centre, while no significant difference with no adverse effects was observed for the second centre (AUC 0.89 vs. 0.90, *p* = 0.95). Contamination of the signal in breast lesions with unsuppressed fat causing a reduction of diagnostic performance of diffusion kurtosis imaging may potentially be counteracted by proposed adapted evaluation methods.

## Introduction

X-ray mammography screening is characterized by a high level of sensitivity in detecting abnormal breast masses, but relatively low specificity in assessing malignancies^1^. This has triggered an increasing interest in additional methods for clarifying ambiguous findings, especially using magnetic resonance imaging (MRI) to avoid the large number of unnecessary biopsies^2–7^. A recent and successful trend has been the use of abbreviated, contrast-agent free protocols based on diffusion-weighted imaging (DWI) and T2-weighted acquisitions, especially in the light of the required short examination times and the possible side effects of Gadolinium containing contrast agents^8^, which is especially relevant when aiming to apply these techniques during the clarification process of routine screening^3,9–11^. While the acquired DWI data is mostly assessed visually in a clinical setting, quantitative multiparametric analysis is gaining increasing attention since it may improve standardization and the still limited specificity of lesion characterization. In this context, diffusion kurtosis imaging (DKI) has been recently introduced in breast DWI analysis yielding a second quantitative parameter, the kurtosis, in addition to the diffusion coefficient^12–19^.

DWI exploits the Brownian motion of water molecules to probe diffusion restrictions such as cell membranes and thus to get insight into tissue structure. In the simplest case of quantitative analysis, the only parameter determined is the apparent diffusion coefficient (ADC), which is usually reduced in tumour tissue. While the ADC is the only measureable parameter for free diffusion due to the Gaussian nature of the diffusion propagator, the displacement distribution of the water molecules is no longer Gaussian when obstacles such as cell membranes are present. This deviation from free diffusion is observable as non-linear decay of the logarithmic signal, which is most apparent at high diffusion weightings *b*. The non-Gaussianity can be quantified by the additional parameter diffusional kurtosis, which is non-zero for restricted diffusion and may yield additional insight into diffusion barriers and tissue heterogeneity^12,20^.

Due to the high content of adipose tissue in the female breast, sufficient fat suppression is crucial for breast DWI, which is often not fully achieved. Residual fat signal may contaminate the signal obtained from lesions as result of T2 blurring, image ghosting, partial volume effects and especially chemical shift artifacts^21^. For high *b-*values, as needed for DKI, this is especially relevant since the water diffusion signal is largely attenuated while the fat signal is only slightly diminished due to the extremely low fat ADC^22–24^. This leads to a background signal level which can distort quantitative parameters obtained in lesions. It can be suspected that a fat-induced elevation of the signal level at high *b-*values can lead to an overestimation of kurtosis values similar to the effect of the background signal induced by Rician noise^12,25^.

The purpose of this investigation was the evaluation of the impact of the residual fat-related signal on the ability of diffusion kurtosis imaging to distinguish between benign and malignant breast lesions initially detected on suspicious screening mammograms. Two possible main sources of fat signal contamination were evaluated here. Firstly, chemical shift is expected to result in a transfer of a background signal level from not fully suppressed adipose tissue to the lesion regions due to the resulting shift in phase encoding direction, while no water signal will be transferred to the lesion region. Due to the low ADC of fat, this signal level is rather independent of the *b*-value. Secondly, partial volume effects may lead to contributions of a mixture of fat and water signal surrounding the lesion to the segmented region. These possible signal contaminations were incorporated into kurtosis evaluation approaches by introducing phenomenological correction terms, which were evaluated using data of MR scanners from different vendors and using different coils and MRI sequences.

## Materials and methods

### Patients

For this study, a retrospective analysis of prospectively acquired data of a bicentric MRI study was performed. Approval was obtained from the Ethics Committee of the Medical Faculty of the University of Heidelberg and the governmental Ethics Committee of the State Medical Association Baden-Württemberg. Experimental studies were conducted complying with all institutional and governmental regulations. The age of all patients was above 18 years. Written informed consent from all patients included in the prospective study was obtained.

198 consecutive patients were analysed of which 105 women (Group A) received examinations at one site and 93 women (Group B) at another study site with a different MRI scanner. The MR datasets included in this study have been previously analysed as part of other evaluations with different objectives^9,13,26–28^, which results in an overlap in the description of patient collective and acquisition methods. However, the influence of residual fat signal and different correction approaches on diagnostic performance has not yet been evaluated in detail so far with the current technical analysis being performed on consecutive datasets with breast lesions visible on *b* = 750 s/mm^2^ or *b* = 1500 s/mm^2^ images of the DWI acquisitions.

All consecutive patients included in this study had received indication for biopsy due to a Breast Imaging Reporting and Data System (BI-RADS) category 4 or 5 finding on X-ray mammography. Each patient, with ambiguous diagnosis after mammography, at first took part in a clarification process incorporating at least one of the commonly accepted diagnostic methods like ultrasound, clinical examination or magnification mammography. After the MRI examination, biopsy was conducted under either ultrasound or stereotactic X-ray guidance using a core needle system.

### Imaging protocol

The MRI scans were acquired in two study centres with MRI scanners from different vendors as previously described^9^. For Group A, a 1.5 T Ingenia MR scanner (Philips, Best, The Netherlands) with a 2-channel breast loop coil combined with elements of the spine coil in the table was used, while data for Group B was acquired with a 1.5 T MAGNETOM Aera (Siemens Erlangen, Germany), equipped with 18-channel breast coil. All patients received a full diagnostic MRI protocol including pre-contrast T2- and T1-weighted imaging (transverse, coronal) and contrast-enhanced T1-weighted imaging. Since, in this study, quantitative DWI-derived parameters were evaluated, only the DWI sequence parameters are presented in detail: *b*-values 0, 100, 750 and 1500 s/mm^2^; in-plane resolution 2.5 × 2.5 mm^2^, slice thickness 3 mm, SPAIR fat suppression. Patients were placed in a prone position. The placement of the breast in the coil was gently supported by soft material, avoiding squeezing. For Group A, a single shot echo planar imaging (EPI) sequence was used with TR 10.6 s, TE 100 ms, phase encoding direction RL, FOV 340 × 400 mm, acquisition bandwidth 2393 Hz/pixel, parallel imaging with SENSE factor 2.5. For Group B, a readout segmented EPI sequence was employed with TR 11.7 s, TE 80 ms, phase encoding direction AP, FOV 480 × 240 mm, acquisition bandwidth 870 Hz/pixel, parallel imaging with GRAPPA (× 2), three readout segments and trace-weighted acquisition. The parameters of the first DWI-sequence were set as close as possible to the second DWI-sequence. However, the selection of the optimal values of the parameters was motivated by the highest visual quality of the diffusion-weighted images obtained on each scanner.

### Volume of interest definition

The delineation of three-dimensional regions of interest (ROIs) was performed in The Medical Imaging Interaction Toolkit (MITK, DKFZ, Heidelberg, Germany) by a postgraduate medical researcher (2 years of experience) with a radiologist (6 years of experience in breast imaging) in consensus.

Using the accompanying information about the index lesion localization from the X-ray screening report and T2-weighted imaging, ROIs were manually defined on the *b* = 1500 s/mm^2^ images or—if not clearly visible for this highest diffusion weighting—on *b* = 750 s/mm^2^, while trying to minimize partial volume effects by using the inner border of the lesion. Additionally, for each patient, a second ROI was drawn on the DWI dataset in an area containing fatty tissue as determined in concordance with the T2-weighted imaging. The oval-shaped fatty tissue area was usually drawn on the contralateral breast, in a mirrored position of the lesion to provide comparable distance to the surface of the coil.

### Calculation of quantitative parameters

The following diffusion and kurtosis evaluation approaches were evaluated to investigate the influence of fat contamination. ${D}_{i}$ ($i=1\dots 5$) and ${K}_{i}$ ($i=2\dots 5$) denote the apparent diffusion coefficient and the apparent kurtosis coefficient obtained by the respective methods.

#### Method 1

The standard apparent diffusion coefficient, termed ${D}_{1}$ here, was obtained using the mono-exponential fit function

1 $$S\left(b\right)={S}_{0}\,{ e}^{-b{D}_{1}}$$

with the two fitting parameters ${D}_{1}$ and ${S}_{0}$, which corresponds to the signal without diffusion weighting. $S(b)$ is the mean signal intensity in the respective lesion ROI.

#### Method 2

For the standard kurtosis fitting approach, the following equation was used:

2 $$S\left(b\right)={S}_{0} \,{e}^{-b{D}_{2}+\frac{1}{6}{b}^{2}{D}_{2}^{2}{K}_{2}}$$

with the modified apparent diffusion coefficient ${D}_{2}$, the apparent kurtosis coefficient ${K}_{2}$, and ${S}_{0}$ as free parameters in the fit.

#### Method 3

This method accounts for possible fat signal contamination due to chemical shift in phase encoding direction, which may transfer unsuppressed fat signal to the lesion ROI. Therefore, a constant signal level was added to Eq. (2), which was determined in fatty tissue:

3 $${S\left(b\right)=S}_{0} \,{e}^{-b{D}_{3}+\frac{1}{6}{b}^{2}{D}_{3}^{2}{K}_{3}}+a\cdot \theta ({b}_{\mathrm{max}})$$

where $\theta \left({b}_{\mathrm{max}}\right)$ denotes the signal measured in the contralateral fat ROI^13^ at the *b* = 1500 s/mm^2^ image. An additional parameter $a$ was introduced to change the background signal level since the shifted residual fat signal may not be completely transferred to the lesion ROI. $a$ was varied from 0 to 1 with 0.1 step size to investigate whether a modified weight of the fat contribution in the modelling of the total signal can increase diagnostic performance.

#### Method 4

The fourth approach is a phenomenological extension of the method initially proposed by Jensen et al.^12^ for accounting for the Rician noise distribution leading to a background signal level in magnitude images, which was later also used in the context of breast imaging^15^. It was introduced here due the close analogy between the constant background signal level that may be introduced by noise and chemical shift. Here, the background noise level $\eta$ (Eq. 16 in^12^) was replaced by $\theta \left({b}_{\mathrm{max}}\right)$:

4 $$S(b)=\sqrt{{\left({S}_{0} \,{e}^{-b{D}_{4}+\frac{1}{6}{b}^{2}{D}_{4}^{2}{K}_{4}}\right)}^{2}+\theta {\left({b}_{\mathrm{max}}\right)}^{2}}$$

where ${D}_{4}$ and ${K}_{4}$ are the respective diffusion and kurtosis coefficients.

#### Method 5

This method considers the partial volume effect caused by the adipose tissue located at the border of the lesion. Since this adipose tissue will contain both, water and fat protons, a dependency on the *b*-value is to be expected. Therefore, a two compartment model was assumed. The absolute magnitude of the fat signal was discarded and signal fraction factor $f$ which controls the weight of the signal attenuation of the standard kurtosis equation compared to the signal decrease in the fat ROI was introduced:

5 $${S\left(b\right)=S}_{0} \left[f{e}^{-b{D}_{5}+\frac{1}{6}{b}^{2}{D}_{5}^{2}{K}_{5}}+\left(1-f\right)\tilde{\theta}(b)\right],$$

where $\tilde{\theta }(b)=\theta (b)/\theta (b=0)$ is the normalized signal decrease in the fat ROI. $f$ was varied from 0 to 1 with 0.1 step size.

Calculation of quantitative parameters was performed using MATLAB (MathWorks, Natick, MA, USA) with a non-linear least square fitting algorithm (trust-region algorithm). First, only the signals for $b>0$ were used; for comparison, calculations were repeated including $S(b=0)$. To suppress outliers, the fit parameters were constrained using $0\le {D}_{i}\le 3.5 \,\mathrm{\mu {m}^{2}/ms}$ and $0\le {K}_{i}\le 3$. These values were chosen according to the fact that the diffusion coefficient of water at body temperature cannot lie outside this range and due to the experimental reports of kurtosis values typically occurring in vivo. Before curve fitting, the average signal of the voxels of the lesion was calculated for each *b*-value yielding the input signal $S(b)$. Additionally, for comparison, parameter calculation was performed for each voxel taking the median value of the resulting ${D}_{i}$ and ${K}_{i}$ parameters afterwards. For voxel-wise calculation, voxels yielding ${D}_{i}$ and ${K}_{i}$ values outside the above-mentioned range were omitted.

### Lesion-to-background ratio (LBR)

A lesion-to-background ratio (LBR) was introduced as a rough measure of conspicuity of the lesion to assess relative magnitude of the possible fat-related signal contamination compared to the lesion signal. It is given by the following equation:

6 $$LBR= \frac{{\mu }_{\mathrm{tumor}}}{{\mu }_{\mathrm{fat}}},$$

where ${\mu }_{\mathrm{tumor}}$ is the average signal in the tumour ROI and ${\mu }_{\mathrm{fat}}$ the average signal in the fat-related ROI, both in the image with *b* = 1500 s/mm^2^. By setting two $LBR$ thresholds, datasets were divided into three subgroups for each study centre cohort with approximately equal number of patients (*LBR* < 1.5; 1.5 ≤ *LBR* < 2; *LBR* ≥ 2). For each subgroup, diagnostic performance was investigated separately for the different evaluation approaches.

### Statistical analysis

Statistical analysis was performed using SigmaPlot (Systat Software Inc., San Jose, CA, USA). The calculated quantitative parameters were tested for significant differences between benign and malignant lesions using the Wilcoxon rank sum test. The diagnostic performance of the obtained parameters was assessed using receiver operating characteristic (ROC) analysis and calculating the area under the curve (AUC). Differences in the ROC curves were investigated using the DeLong method. For *p* values smaller than 0.05, the differences were considered to be significant. Logistic regression was performed to combine the ${D}_{i}$ and ${K}_{i}$ values for prediction of malignancy for methods 2–5. The subgroups obtained for the different $LBR$ thresholds were analyzed separately.

## Results

**Figure 1:**
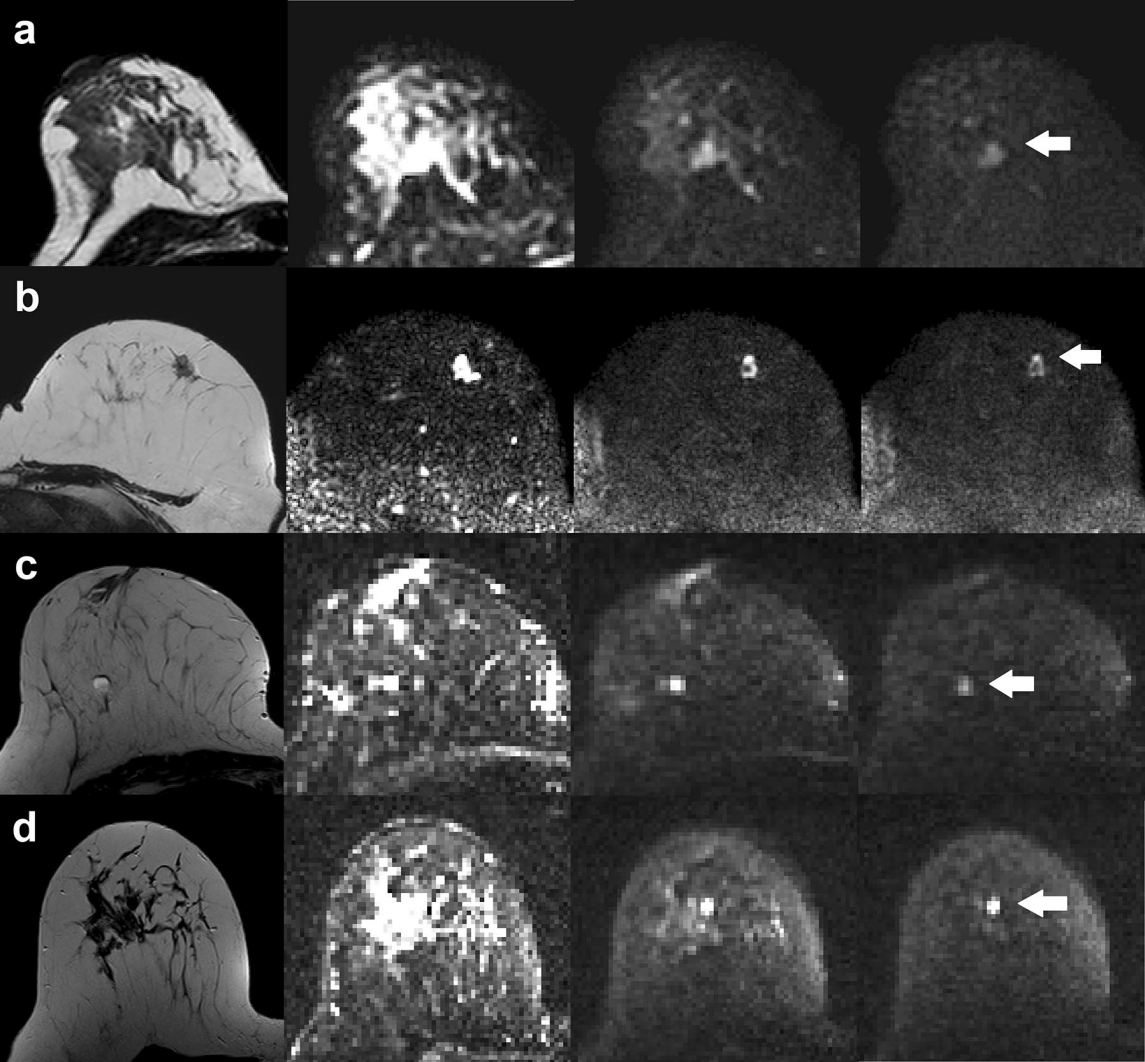
Examples of T2-weighted images (column on the left) and transversal DW images (*b*-values 0, 750 and 1500 s/mm^2^, respectively) of 4 patients. White arrows depict location of the lesions. (**a**) Benign lesion, cohort A. (**b**) Malignant lesion, cohort A. (**c**) Benign lesion, cohort B. (**d**) Malignant lesion, cohort B.

Histopathology revealed 45 benign and 60 malignant lesions in Group A and 32 benign and 61 malignant lesions in Group B. The median number of voxels of the ROIs for benign lesions was 11 and 14 in Group A and Group B, respectively. It was 21 and 25 for malignant lesions, respectively. The median number of voxels of fat-ROIs was 51 in Group A and 47 voxels in Group B. Figure 1 depicts representative DWI scans for different *b*-values and T2-weighted images for benign and malignant findings from the two study centres.

The results of methods 1–4 are presented separately from method 5, where the additional signal fraction factor $f$ was introduced.

### Comparison of standard methods to fat-corrected approaches (methods 1–4)

**Figure 2:**
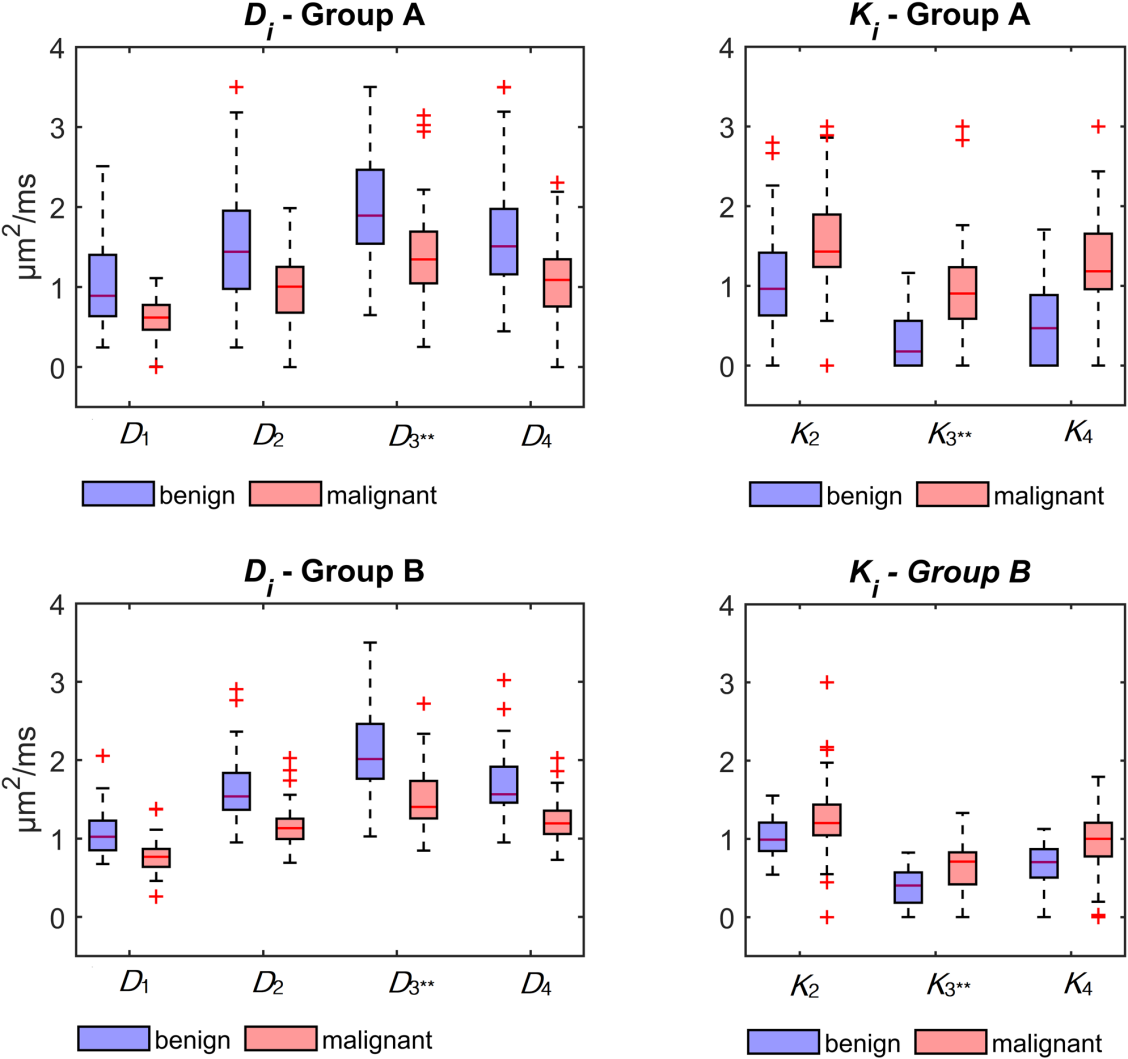
Boxplots of the diffusion coefficients ${D}_{i}$ and kurtosis coefficients ${K}_{i}$ for methods 1–4 for benign (blue) and malignant (red) lesions. Significantly lower ${D}_{i}$ and higher ${K}_{i}$ values were observed for malignant lesions. Method 3^**^ presents the results for the maximal fat signal contribution (*a* = 1).

First, the results related to methods 1–4 are presented, which assume contamination with a constant fat-related signal for each *b-*value. To start with, the results for method 3 are presented for the maximal fat signal contribution (*a* = 1), denoted by two asterisks. The apparent diffusion coefficients ${D}_{i}$ and apparent kurtosis coefficients ${K}_{i}$ are graphically shown in boxplots in Fig. 2. These values were estimated for 3 *b*-values by fitting to the mean signal.

For all methods, significantly lower ${D}_{i}$ values were observed in malignant lesions compared to benign ones (*p* < 0.001). On the other hand, significantly higher kurtosis values ${K}_{i}$ were observed for malignancies (*p* < 0.001) with all methods, as expected. When introducing the fat correction for methods 3^**^ and 4, a tendency for increasing ${D}_{i}$ and decreasing ${K}_{i}$ can be noticed. A considerably broader dispersion of the values can be observed for Group A for both, ${D}_{i}$ and ${K}_{i}$.

The AUC values obtained in the ROC analysis of single parameters are displayed in Table 1.

**Table 1:** Area under the ROC curves for diffusion and kurtosis parameters calculated for methods 1–4 for all patients (95% confidence intervals in parenthesis).

Method	Parameter	
	*D*_*i*_	*K*_*i*_
1	0.77 (0.70–0.84)	–
2	0.78 (0.70–0.85)	0.70 (0.63–0.78)
3**	0.79 (0.72–0.85)	0.75 (0.68–0.82)
4	0.79 (0.72–0.85)	0.76 (0.69–0.83)

**Results for the maximal fat signal contribution (*a* = 1).

For the diffusion coefficient, a trend towards higher AUC values could be observed for methods 3^**^ and 4, which was, however, not significant. Considering diffusional kurtosis, the AUC value was higher for method 4 compared to method 2 (0.76 vs 0.70, *p* = 0.049).

**Figure 3:**
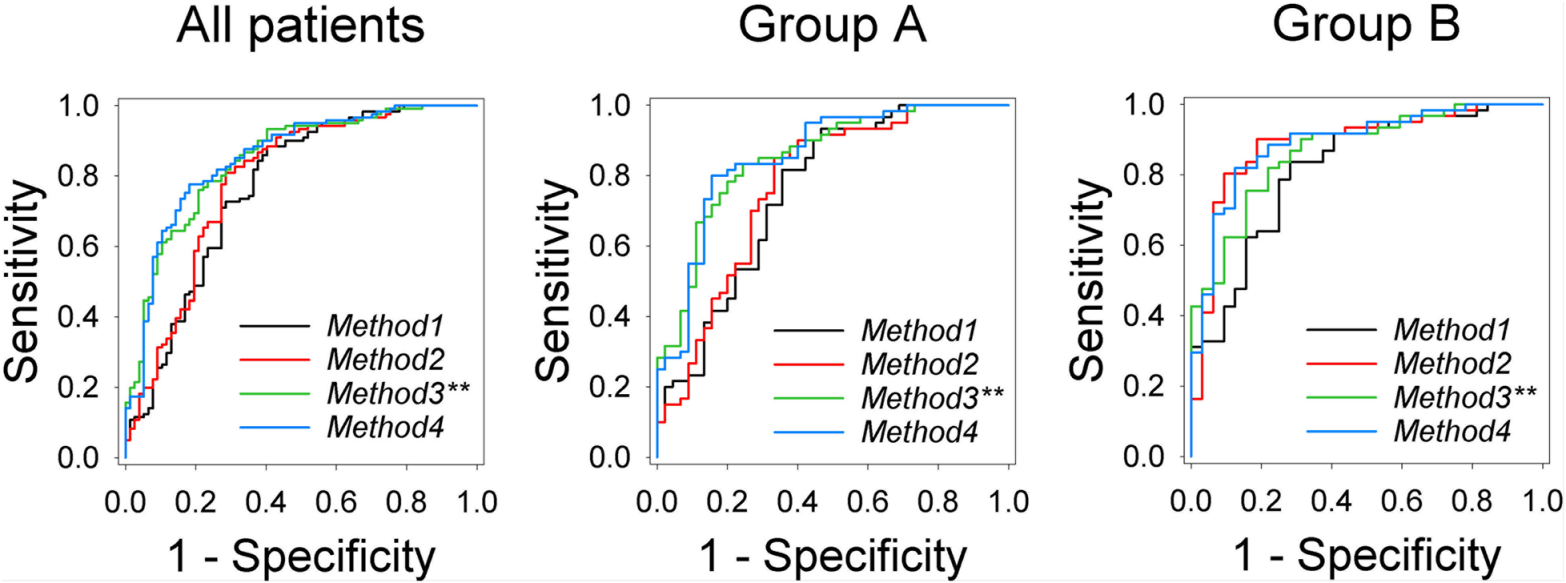
ROC curves for methods 1–4 for all patients, Group A, and Group B respectively. Method 3^**^ and method 4 discriminate best between benign and malignant lesion. In the individual analysis, the superiority of the adapted models can be seen only in Group A. Method 3^**^ corresponds to the results for the maximal fat signal contribution (*a* = 1).

The ROC curves obtained for methods 1–4 using logistic regression with the two predictors ${D}_{i}$ and ${K}_{i}$ are shown in Fig. 3, the AUC values are stated in Table 2a.

**Table 2:** Area under the ROC curves for logistic regression with ${D}_{i}$ and ${K}_{i}$ as predictors calculated for methods 1–4 for all patients and the two study centres separately, with 95% confidence intervals in parenthesis.

Method	All	Group A	Group B
**(a) Parameters estimated for 3 b-values by fitting to the mean signal**			
1	0.77 (0.70–0.84)	0.76 (0.66–0.86)	0.82 (0.73–0.91)
2	0.79 (0.72–0.86)	0.77 (0.68–0.87)	0.89 (0.82–0.97)
3**	0.85 (0.79–0.90)	0.85 (0.78–0.93)	0.87 (0.80–0.94)
4	0.85 (0.80–0.91)	0.86 (0.78–0.93)	0.89 (0.82–0.96)
**(b) Parameters estimated for 4 b-values by fitting to the mean signal**			
1	0.78 (0.70–0.85)	0.76 (0.67–0.86)	0.83 (0.74–0.92)
2	0.79 (0.72–0.86)	0.78 (0.69–0.87)	0.85 (0.77–0.93)
3**	0.84 (0.78–0.89)	0.85 (0.78–0.92)	0.85 (0.78–0.93)
4	0.85 (0.80–0.91)	0.86 (0.79–0.93)	0.87 (0.80–0.94)
**(c) Parameters estimated for 3 b-values by voxel-by-voxel fitting**			
1	0.78 (0.71–0.85)	0.78 (0.68–0.87)	0.82 (0.73–0.92)
2	0.80 (0.73–0.87)	0.80 (0.70–0.89)	0.84 (0.75–0.93)
3**	0.84 (0.79–0.90)	0.85 (0.78–0.92)	0.87 (0.78–0.95)
4	0.84 (0.79–0.90)	0.83 (0.75–0.91)	0.89 (0.83–0.96)

(a) Calculations for 3 *b*-values and the mean signal, (b) calculations for 4 *b*-values and the mean signal, (c) calculations voxel by voxel for 3 *b*-values.

**Results for the maximal fat signal contribution (*a* = 1).

For the entire study collective, method 4 (AUC 0.85) accounting for residual fat signal performed significantly (*p* < 0.015) better than methods 1 (AUC 0.77) and 2 (AUC 0.79); higher AUC values could be observed for method 3^**^ (AUC 0.85) when compared to method 1 (*p* = 0.020), and method 2 (*p* = 0.068). The significant increase in AUC could also be observed in Group A alone for method 4 (*p* < 0.035). The changes in AUC of all methods for Group B were not significant (*p* > 0.068). Especially for Group B, the AUC for method 1 using the diffusion coefficient alone was lower than for the other methods, but the differences were not statistically significant.

The analysis of the influence of the stepwise increase of the fat signal incorporated into the method 3, taking into account all the patients, revealed that the AUC value was increasing with the fraction of the fat signal. A similar tendency holds for Group A, whereas the AUC changes are very small for Group B (Table 3).

**Table 3:** AUC values for the logistic regression with parameters obtained for an increasing fraction of the fat signal incorporated into the fitting process.

	a = 0.1	a = 0.2	a = 0.3	a = 0.4	a = 0.5	a = 0.6	a = 0.7	a = 0.8	a = 0.9	a = 1
All	0.79	0.80	0.81	0.81	0.82	0.83	0.84	0.85	0.85	0.85
Group A	0.78	0.79	0.80	0.80	0.81	0.83	0.85	0.85	0.85	0.85
Group B	0.89	0.89	0.89	0.90	0.90	0.90	0.89	0.89	0.88	0.87

Increasing *a* value indicates higher fraction of the fat signal.

The above-presented results were obtained excluding the $S(b=0)$ values. In Table 2b, the results for logistic regression with ${D}_{i}$ and ${K}_{i}$ calculated with all *b-*values are shown. AUC values are very similar to Table 2a with the highest AUC value being 0.85 for method 4; for group A, the significantly better performance of method 4 compared to methods 1 and 2 could be again observed.

The values obtained for voxel-by-voxel calculation, again using only images with *b* > 0, did also reveal the highest AUC for method 3^**^ and method 4 (Table 2c). The results did not significantly differ from the ones presented in Table 2a obtained with ROI-based signal averaging.

Table 4 reports specificity for a sensitivity fixed at 95% for ROC for multiple logistic regression with coefficients estimated for 3 *b*-values by fitting to the mean signal. For the entire patient collective, method 4 showed the highest specificity (52%) compared to 44% for method 1, 45% for method 3^**^ and 38% for method 2. Method 4 also resulted in the highest specificity for the individual groups A and B with 58% and 50%, respectively.

**Table 4:** Specificity for 95% sensitivity for logistic regression models with ${D}_{i}$ and ${K}_{i}$ as predictors calculated with methods 1–4 (95% confidence intervals in parenthesis).

	All patients	Group A	Group B
Method 1	44% (33–56%)	38% (24–53%)	44% (26–62%)
Method 2	38% (27–49%)	33% (20–49%)	47% (29–65%)
Method 3**	45% (34–57%)	49% (34–64%)	41% (24–59%)
Method 4	52% (40–63%)	58% (42–72%)	50% (32–68%)

**Results for the maximal fat signal contribution (*a* = 1).

### Lesion to background ratio (LBR) subgroups

The division of patients into subgroups depending on *LBR* in order to investigate the performance of the methods for different lesion conspicuity is presented in Table 5.

**Table 5:** Descriptive statistics of the *LBR* values for the subgroups obtained by *LBR* thresholds.

Group A	mean ± std	min	max	benign A	malignant A
*LBR* < 1.5	1.21 ± 0.16	0.86	1.47	22	13
1.5 ≤ *LBR* < 2	1.74 ± 0.15	1.54	1.98	13	13
*LBR* ≥ 2	2.78 ± 0.91	2.03	6.41	10	34

Group B	mean ± std	min	max	benign B	malignant B
*LBR* < 1.5	1.28 ± 0.14	0.93	1.49	16	11
1.5 ≤ *LBR* < 2	1.77 ± 0.14	1.51	1.99	10	18
*LBR* ≥ 2	2.50 ± 0.44	2.00	3.74	6	32

As expected, the fraction of malignant lesions increases for higher *LBR*, since a similar signal attenuation is expected in the fat ROI for benign and malignant findings while higher DWI signals on *b* = 1500 s/mm^2^ are expected for malignancy. All lesions in cohort B that were not visible on *b* = 1500 s/mm^2^ belonged to the lowest *LBR* group. In cohort A, this held true for the majority of lesions. In both cohorts, benign cases are very frequent in the subgroup with low *LBR*.

Table 6 shows the AUC values for the *LBR* subgroups. In the subgroup with the lowest *LBR* values, method 4 performs best for the entire cohort but also for group A; but the differences were not significant. This trend seems to be preserved for the middle *LBR* group, however less pronounced, while becoming not observable for *LBR* ≥ 2.0. The analysis confirms that there does not seem to be a significant influence of the fat correction terms for study group B.

**Table 6:** AUC for logistic regression with ${D}_{i}$ and ${K}_{i}$ as predictors for the different LBR subgroups.

	All	Group A	Group B
Method	*LBR* < 1.5		
1	0.80 (0.69–0.91)	0.81 (0.66–0.95)	0.82 (0.62–1.03)
2	0.78 (0.67–0.90)	0.78 (0.62–0.94)	0.88 (0.71–1.04)
3**	0.80 (0.69–0.91)	0.85 (0.72–0.98)	0.74 (0.54–0.95)
4	0.82 (0.71–0.93)	0.87 (0.75–0.99)	0.85 (0.69–1.01)
Method	1.5 ≤ *LBR* < 2.0		
1	0.78 (0.64–0.92)	0.74 (0.54–0.94)	0.92 (0.79–1.05)
2	0.83 (0.71–0.95)	0.78 (0.58–0.98)	0.92 (0.82–1.02)
3**	0.83 (0.71–0.96)	0.80 (0.61–0.99)	0.94 (0.87–1.02)
4	0.83 (0.71–0.96)	0.78 (0.58–0.97)	0.94 (0.85–1.03)
Method	*LBR* ≥ 2.0		
1	0.78 (0.65–0.91)	0.81 (0.65–0.96)	0.82 (0.68–0.97)
2	0.83 (0.72–0.94)	0.88 (0.76–1.00)	0.83 (0.64–1.02)
3**	0.80 (0.68–0.92)	0.84 (0.69–0.98)	0.81 (0.63–1.00)
4	0.82 (0.70–0.93)	0.84 (0.70–0.99)	0.82 (0.63–1.02)

**Results for the maximal fat signal contribution (*a* = 1).

### Variation of fat signal contributions depending on *b*-value (method 5)

For methods 5, varying the relative weight $f$ of the signal contributions to the fit function, the AUC values obtained using logistic regression are given in Table 7 for ten different $f$-values. The best results were obtained for $f=1$, which essentially corresponds method 2 without any additional term accounting for the fat contribution. Therefore, method 5 is inferior to methods 3 and 4.

**Table 7:** AUC values for logistic regression models with parameters calculated from equations with fractionated fat contribution (methods 5).

	f = 0.1	f = 0.2	f = 0.3	f = 0.4	f = 0.5	f = 0.6	f = 0.7	f = 0.8	f = 0.9	f = 1
All	0.59	0.61	0.63	0.69	0.74	0.75	0.77	0.78	0.79	0.79
Group A	0.67	0.68	0.69	0.70	0.70	0.71	0.74	0.75	0.77	0.77
Group B	0.77	0.78	0.80	0.81	0.83	0.82	0.84	0.86	0.88	0.89

Higher *f*-values correspond to higher contributions from the signal in lesion and lower from fatty tissue area. For *f* = 1, no fat correction is applied.

## Discussion

The aim of the study was the evaluation of the ability to introduce phenomenological parameters into the diffusion kurtosis evaluation equations to reduce the effect of unsuppressed residual fat signal contributions. In this context, two main sources of possible fat contamination were evaluated: chemical shift and partial volume effects. To account for chemical shift related fat contamination, a constant background signal level obtained from fat regions was added to the standard kurtosis evaluation equation and additionally varied by a constant factor. Additionally, the method introduced initially by Jensen et al. to account for signal noise contributions was evaluated after phenomenologically replacing the noise level in the air outside the patient’s body by the residual fat signal level^12,13^. The proposed methods were motivated by the observation that an elevated background signal level can lead to artificially increased kurtosis values, especially in lesions with low conspicuity, which can be counteracted by introducing a corresponding background signal term in the fitting equations^13^. Overall, methods 3 and 4 exhibited the highest AUC values, which were significantly better than the conventional methods 1 and 2, with the trend of best specificity at 95% sensitivity for method 4. Particularly, a similar approach to the one developed by Jensen et al. was previously employed by Iima et al., however to account for the noise level of the signal and not to account for fat signal^15^.

In our study, as expected, using a monoexponential fit (method 1) for the conventional ADC evaluation approach yields lower values for the diffusion coefficient compared to the DKI evaluation (method 2), since the kurtosis-related curvature of the signal with increasing *b*-value is included into the ADC calculation. When including the fat correction terms, the expected decrease of the kurtosis values occurs, since the artificial curvature introduced into the signal by a background signal level is compensated giving more accurate kurtosis values. Inclusion of *b* = 0 measurements into the evaluation does not seem to have significant impact on the results, which is reasonable due to low fraction of total acquisition time spent for acquiring these images. Voxel-by-voxel parameter calculation as opposed to ROI based signal averaging did not significantly change the results as well.

The evaluation of the methods 1 to 4 for different relative lesion signal intensity on *b* = 1500 s/mm^2^ compared to the fat signal regions indicates that lesions with low visibility on images with high *b*-value may be the affected the most by residual fat signal contamination.

Introducing the additional parameter $a$ to adjust the fat signal level in method 3 does not seem to be desirable: No improvement compared $a=1$ is observed and an additional parameter is introduced which has to be chosen.

In contrast to method 3 and 4 assuming chemical shift as the main source of signal contamination, method 5 assuming partial volume effects does not yield an improvement, which suggests that chemical shift of residual fat signal may indeed be the most relevant effect.

For the different study groups A and B, which were acquired on MR scanners from different vendors, the results were significantly different. The improvement in diagnostic performance for the methods including fat correction was only observed for group A, while the standard DKI approach yielded very good results for group B. This implies that fat contamination seems a less pronounced problem for this study cohort. This may be due to the use of a segmented EPI sequence as opposed to a single-shot approach, differences in the details of the fat suppression techniques or the image postprocessing, which applied considerably more changes to the images for group A. This shows that the proposed correction methods improve the results in multi-centre setting with a heterogeneous scanner setup. The aim of this study was not the use of identical scanners and sequence parameters but rather to demonstrate that—in a heterogeneous setting where each scanner with different technical properties was optimized for optimal image quality—quantitative kurtosis based evaluation can be performed with high quality. In the future, a more detailed evaluation of the observed differences in MR sequence details and their impact of the background signal level possibly hampering DKI evaluations may be an important step. These differences also show the necessity for further standardization of DWI protocols and the corresponding quality control to establish comparable quantitative diffusion-based biomarkers for different study centres, which is of high importance with regards to use such imaging biomarkers for precision medicine in diagnostic and supervision of therapy.

The importance of a sufficient fat suppression in breast DWI acquisitions for achieving a good diagnostic performance has been previously observed^29^ and is stressed by our results. Several studies have proposed the superiority of the kurtosis diffusion approach over the pure Gaussian evaluation approach in the diagnostic accuracy of breast lesions^14,17,30,31^. Our quantitative values for diffusion coefficient and kurtosis are in a similar range, which is in line with a recent study on another patient population^32^. The results of our study suggest that diagnostic performance of breast DKI can be further increased by accounting for residual fat signal, depending on details of the MR acquisition technique.

It has to be stressed that there is no direct relation between the parameters introduced in the fitting equations and the volume fractions of the underlying tissue compartments. Especially, no biophysical modelling of tissue compartments was performed to calculate the expected resulting signal, which has, for example, been performed for prostate imaging^33^, and which may yield additional insights. However, this is a difficult endeavour since the fat volume fraction in tissue is not the parameter mainly governing the fat signal contribution. It is mainly determined by the efficiency of the fat suppression technique which is influenced by many parameters in a non-trivial way, such as field homogeneity, patient geometry and the choice of frequency and bandwidth of the fat excitation pulses. This lack of a direct relation to underlying biophysiology may hamper the applicability of the proposed techniques on other MRI scanners or using different sequence implementations. Further, introducing an additional fat ROI, which was manually positioned here, may introduce an additional source of error compared to the standard approach (method 2). This may undermine stability for clinical routine applications.

Furthermore, it has to be noted that several noise correction algorithms^12,15,34,35^ for DKI have been proposed, which have, however, not found widespread application in clinical studies to date. This may result from the increased complexity of the evaluation approaches which could also introduce additional variations. Moreover, the observations presented in this work are only based on two MRI scanners, which may raise questions regarding generalizability. This highlights that further evaluations regarding the possible merit of the methods proposed in this study are needed to assess their actual clinical value.

Limitations include the restricted number of patients, especially for the analysis of subgroups with different lesion visibility. Additionally, the method originally proposed by Jensen et al. including the background signal level in a noise region outside the patient could not be evaluated since the background signal was masked by a post-processing algorithm on the MR scanner for Group A^12,15^. Further, due to the limited acquisition time, the number of *b*-values as well as the choice of the highest *b* value may not be optimal for DKI analysis^36^. For example, in other studies, higher *b* values and a larger number of them were used^14,16,17^. Nonetheless, the primary objective of this study was to create a shortened DKI protocol applicable in the clinical routine. Therefore, the choice of the relatively low maximum *b*-value of 1500 s/mm^2^ is a compromise between the need for a longer acquisition time for higher *b*-values due to decrease in SNR and the need for high *b*-values for sufficiently reliable kurtosis estimations. On the other hand, the use of high *b-*values may lead to distortions of the obtained apparent kurtosis values due to contributions of higher orders of the cumulant expansion of the signal^37^; another group used even smaller maximal *b*-value of 1300 s/mm^2^ for characterizing breast lesions^19^. In addition, details of the image post-processing performed for group A were not known; saving the k-space raw data to reconstruct images without additional post-processing steps may be a viable approach to investigate the effect of this procedure. Also, the effect of the different sequence parameters for the two cohorts has not been evaluated in detail. For instance, the different echo times used on the two MRI systems may have an impact on the fat signal fractions. Assuming typical T1 and T2 values^38,39^ for fatty tissue (T1 = 264 ms, T2 = 58 ms), benign tissue (T1 = 1050 ms, T2 = 89 ms) and malignant tissue (T1 = 876 ms, T2 = 75 ms), an increase in TE from 80 ms (group B) to 100 ms (group A) would lead to 29% signal decrease in adipose tissue, 20% in benign lesions, and 23% in malignant tissue, according to $S \sim (1-\mathrm{exp}(-TR/T1)) \mathrm{exp}(-TE/T2)$ and the sequence parameters used in the two groups. This estimation suggests that group B should be more prone to fat signal contamination when considering TE only, which, however, contradicts the observation that group B did profit less from the fat correction approaches (methods 3–4). It can thus be assumed that other sequence properties such as differences in the implementation of the fat suppression techniques and the use of segmented versus single-shot EPI sequences are more relevant.

In conclusion, method 4 seems to be the most desirable approach. The inclusion of the additional correction term leads to a better kurtosis-based differentiation between malignant and benign breast lesions. It did improve the results in one study centre, but it did not decrease the diagnostic performance in the second one. While needing further validation regarding generalizability to other imaging settings and stability in a clinical context, accounting for background signal levels originating from unsuppressed fat signal transferred into lesion ROIs via chemical shift may be a further step towards the application of DKI-based MR-mammography for selected clinical indications.

## Data Availability

The quantitative evaluation datasets analysed in this study are not publicly available due to legal restrictions following the General Data Protection Regulation (ger.: DSGVO), but are in principle available from the corresponding author on reasonable request if complying with all legal requirements.
